# Limited predictive value of admission time in clinical psychiatry

**DOI:** 10.1186/s12913-020-05806-1

**Published:** 2020-11-13

**Authors:** Peter M. Kreuzer, Stefan Günther, Jorge Simoes, Michael Ziereis, Berthold Langguth

**Affiliations:** Department of Psychiatry and Psychotherapy, University of Regensburg, Universitätsstrasse 84, 93053 Regensburg, Germany

**Keywords:** Admission times, Clinical psychiatry, Diagnostic groups, Circadian rhythms, Mental crisis

## Abstract

**Background:**

A large proportion of admissions to psychiatric hospitals happen as emergency admissions and many of them occur out of core working hours (during the weekends, on public holidays and during night time). However, very little is known about what determines admission times and whether the information of admission time bears any relevance for the clinical course of the patients. In other words, do admission times correlate with diagnostic groups? Can accumulations of crises be detected regarding circadian or weekly rhythms? Can any differences between workdays and weekends/public holidays be detected? May it even be possible to use information on admission times as a predictor for clinical relevance and severity of the presented condition measured by the length of stay?

**Methods:**

In the present manuscript we analyzed data derived from 37′705 admissions to the Psychiatric District Hospital of Regensburg located in the Southern part of Germany covering the years 2013 to 2018 with regard to ICD-10 diagnostic groups and admission times. The hospital provides 475 beds for in-patient treatment in all fields of clinical psychiatry including geriatrics and addiction medicine.

**Results:**

Several core questions could be answered based on our analysis: 1st Our analysis confirms that there is a high percentage of unheralded admissions out of core time showing broad variation. 2nd In contrary to many psychiatrists’ misconceptions the time of admission has no relevant impact on the length of stay in the hospital. 3rd The predictive value of admission time regarding the allocation to ICD-10 diagnostic groups is low explaining only 1% of variability.

**Conclusions:**

Taken together, our data reveal the enormous variation of admission times of psychiatric patients accounting for the need of adequate and consistent provision of personnel and spatial resources.

## Background

Psychiatric conditions are frequently associated with disrupted circadian rhythms [1]. Abnormal sleep patterns experienced by these patients are largely assumed to be either disease-related or due to the lack of day structure. Medication may also play a role. It has been hypothesized that brain disorders and abnormal sleep may ground on a common mechanistic origin and many pathologies found in psychiatric diseases may arise from a destabilization of sleep mechanisms [1]. Disruptions of circadian rhythms have even been suggested as potentially causal factors in bipolar disorder (BD) [2] as indicated by circadian alterations even in drug naïve BD patients independently of their mood status [3] although consistent changes in sleep patterns have not been detected in BD patients [3].

Moreover, as every single reader may remember from personal “dark days”, pondering, pain and concerns are perceived the most during night when little distraction is available and mindfulness is focused on ourselves. Therefore, it seems plausible, that psychiatric conditions tend to exacerbate overnight [4] and it may be speculated that patients seek help in order to avoid these critical hours. Admissions after core working hours may be discerning and potentially hazardous from two perspectives: Firstly, the quality of care of the patient might be affected by the lack of personnel and adequate institutional resources in night hours. This factor might even exert a stronger influence in psychiatric care than in other medical fields due to the high relevance of “speaking medicine” and social interactions and the lack of apparative components (that are typically more or less independent from the patients’ and investigator’s alertness and mood state). Secondly, a high number of over-night admissions strongly affect the working conditions and subsequently the quality of life of the personnel on duty. Therefore, from an epidemiological and management related point of view the question to which extent psychiatric diseases tend to exacerbate overnight and bring patients to seek hospital admission out of core working hours is of great interest regarding options to redeploy personnel and introduce new solutions for providing adequate service.

However, empirical data examining this theoretical background are scarce. Most available studies focused on chronotypes and specific diagnoses using circadian rhythm markers such as cortisol and actometry [3]. In contrast, data regarding hospital admission times in psychiatric units were (in contrast to admission times and quality of care in somatic emergency units) very scarcely systematically assessed. Therefore, we analyzed in detail admission times of more than 37′000 psychiatric admissions during a 6 years’ period in a Psychiatric District Hospital in the Southern part of Germany (Regensburg, Germany). Our main research objectives were:

1. Which proportion of admissions in a psychiatric hospital does occur out of core time (e.g. during night hours and/or weekend/public holidays)? This is considered a vital piece of information for the development of innovative deployment concepts in order to provide adequate personnel resources in psychiatric care.
2. Do admissions out of core time (night/Saturday/Sunday/public holiday) indicate a higher degree of disease severity which may be measured by the surrogate parameter of a longer duration of stay in the hospital? This would represent an additional parameter complementing clinical evaluation regarding disease severity in psychiatric conditions.
3. May the factor “admission time” (potentially in combination with demographic factors such as age and gender) serve as a predictor for the ICD-10 diagnostic group of the patient? This information would in fact help to amend clinical evaluations and provide adequate and disease-specific supply of psychiatric care on an individual level.

## Methods

In the present manuscript we analyzed data derived from 37′705 admissions to the Psychiatric District Hospital of Regensburg located in the Southern part of Germany covering the years 2013 to 2018 with regard to admission times, diagnostic grouping and length of stay. The hospital provides 475 beds for in-patient treatment in all fields of clinical psychiatry including geriatrics and addiction medicine. The Psychiatric District Hospital of Regensburg serves a population of nearly 700′000 people as exclusive, single provider of in-patient psychiatric treatment.

All data were saved and processed in at first step pseudonymized and later anonymized form using excel 2018 and SPSS (IBM Corp. Released 2017. IBM SPSS Statistics for Windows, Version 25.0. Armonk, NY: IBM Corp) software packages. All statistical analyses were conducted with R statistical software (version 3.4.4, R Development Core Team, 2015) [5], alongside the “tidyverse” package (Wickham, 2017) [6] and the “caret” package [7].

For analyzing the relationship between time of admission and diagnosis we performed Linear Discriminant Analyses (LDAs) for predicting the diagnosis from gender and age alone and from gender, age and time of admission. To do so, a train/test split was used where 85% of the data were used to train the model and the remaining 15% were used to test the model.

As we could not determine whether data was missed at random no imputation method was used. Thus, missing observations in pairwise comparisons were excluded from the analysis. Categorical items were dummy-coded as a requirement of the statistical software. The variance inflation [8] factor suggested that none of the variables used in the analysis suffered from large multicollinearity. All statistical tests were unadjusted for multiple comparisons using a significance threshold of 5% if not otherwise mentioned. Data in the text is given as mean ± standard deviation.

No ethics approval was necessary for the conducted analyses presented in this manuscript due to the fact, that according to German laws and international guidelines such retrospective study without any study-related clinical intervention or use of patients’ personal data does not have to be submitted to the ethics committee and is free of ethic-approval obligation (by note of the Ethics Committee of the Faculty of Medicine, 02/05/2013; Code 13–180-0098).

## Results

**Fig. 1:**
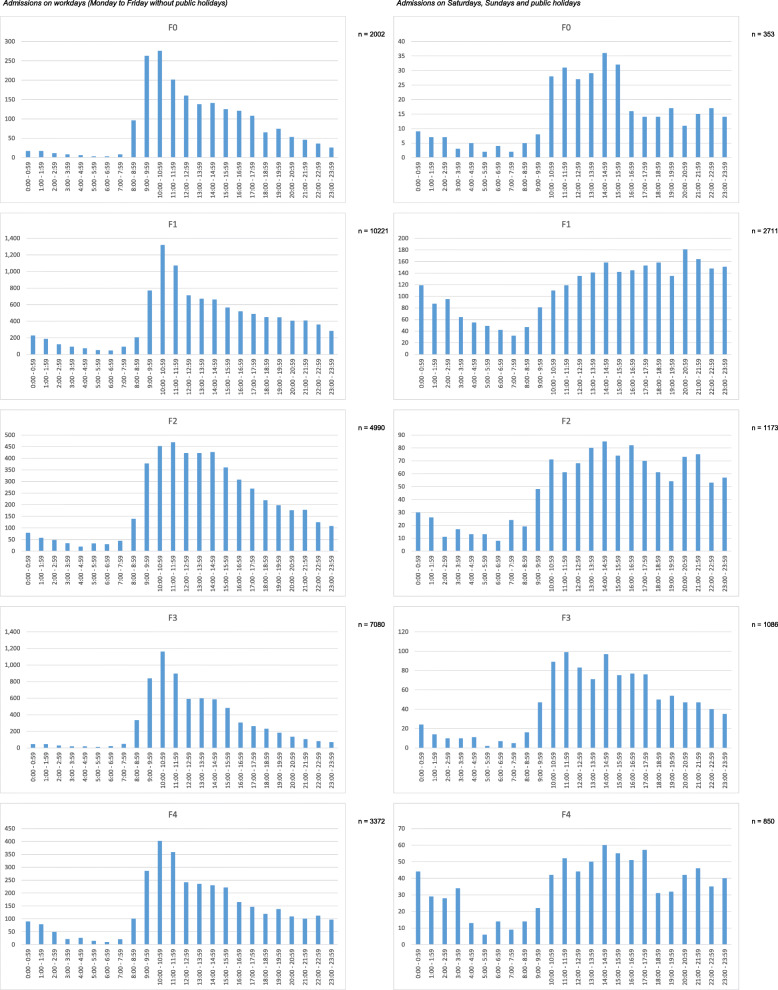

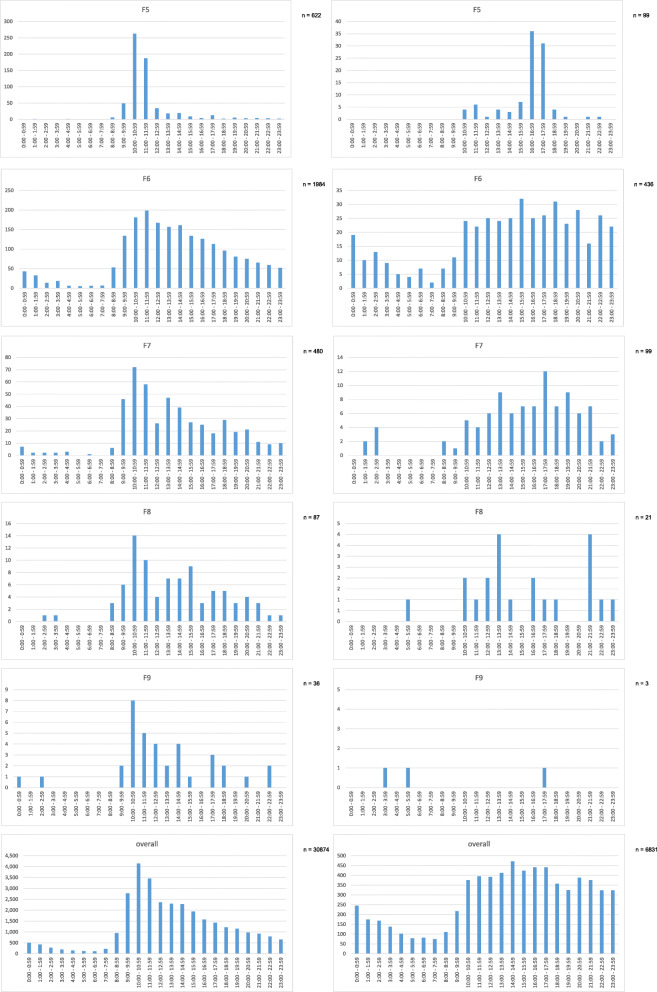
Admissions between 2013 and 2018 grouped by F-diagnoses. Admissions on workdays see graphics left, admissions out of workdays (Saturday, Sunday, public holidays) see right sight. n numbers indicate the amount of cases included in the graphical analysis

Descriptive data indicating diagnostic groups and admission times are presented in Fig. 1. The included plots show admission times concerning the main diagnostic chapters of ICD-10 F-categories. Admissions on working days are displayed left, admissions on weekends and public holidays on the right-hand side. In some diagnostic categories relevant differences concerning working day and weekend/holiday rhythms can be detected on a descriptive level. For example, patients with F1-category show a relevant increase of admissions on weekends/holidays during 10:00 pm and 03:00 am, whereas on working days the early morning hours are far less popular to seek admission for this patient category. Similar tendencies can be observed for diagnostic categories F4 and F6. For the remaining diagnostic groups, no relevant differences between admission times on working days and weekend/holidays could be detected.

Concerning all 37′705 admissions (regardless of diagnostic sub-categories) a similar pattern as in group F1, F4 and F6 could be detected: on working days only 24.9% of admissions occur out of core working time (08.30 am to 05:00 pm), on weekends/public holidays this percentage rises up to 46.2%. Even on working days 18.5% of admissions occur after 05:00 pm and before 00:00 pm with a steep decline towards midnight. On weekends and public holidays this fraction of admissions (05:00 pm to 00:00 pm) is 30.6%. On working days, a “main time” of admissions between 08:00 am and 11:00 pm can be observed; similar structures on weekends/public holidays are missing with admission counts rising from 09:00 am to a stable level until midnight.

**Fig. 2:**
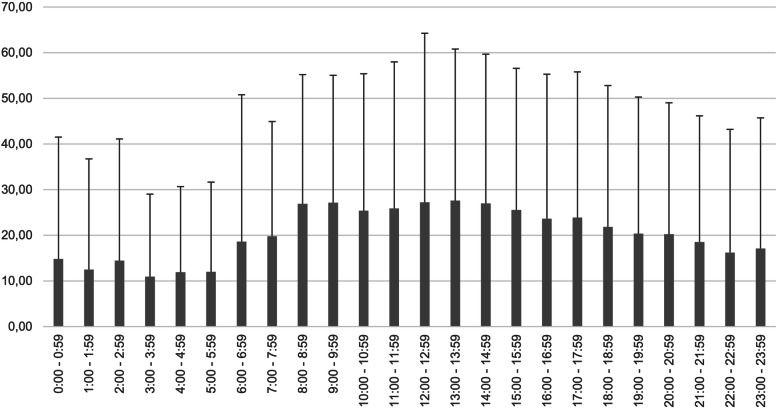
Mean duration of stay depending on time of admission (in days) (mean + standard deviation)

Table 1 shows the results of the linear regression model with the predictors age, gender, and time of admission on length of hospital stay (R2 = 0.03, F (25, 35,278) = 39.4, *p* < 0.001). We observed that different admission times were associated with different staying in the hospital, which can be appreciated by the coefficients during different hours of the day. For instance, admission at the hospital between 13:00 and 13:59 was associated with an increased length of stay of 13.26 days compared to patients who were admitted between 00:00 and 00:59, after controlling for all the covariates in the model. We observed a similar R2 from a multiple linear regression with length of hospital stay as dependent variable, and with the predictors age and gender, but without the time of admission (Supplementary table 1; R2 = 0.01, F (2, 35,301), *p* < 0.001), indicating that the accuracy of both models was similar. Figure 2 shows the mean duration of stay referring to the time of admission.

**Table 1:** Linear regression with the predictors “age”, “gender”, and “time of hospital admission”, and with the dependent variable “length of stay”. Dummy codes were used for the categorical items, with the male gender and the time period between 00:00–00:59 being used as reference. * *p* values < 0.05; ** *p* values < 0.01; *** *p* values < 0.001

	Estimate	Std Error	T value
Intercept	14.45	1.17	12.31***
Gender (Female)	3.74	0.31	11.83***
Age	−0.03	0.01	−3.03**
01:00–01:59	−2.38	1.69	−1.40
02:00–02:59	− 1.85	1.84	− 0.38
03:00–03:59	−3.84	2.04	−1.87
04:00–04:59	−2.84	2.23	− 1.26
05:00–05:59	− 2.79	2.46	−1.13
06:00–06:59	4.01	2.23	1.63
07:00–07:59	5.28	2.13	2.47*
08:00–08:59	12.28	1.45	8.43***
09:00–09:59	12.68	1.24	10.14***
10:00–10:59	10.88	1.20	9.02***
11:00–11:59	11.28	1.22	9.24***
12:00–12:59	12.76	1.26	10.11***
13:00–13:59	13.26	1.26	10.49***
14:00–14:59	12.62	1.26	9.99***
15:00–15:59	13.26	1.28	8.51***
16:00–16:59	9.16	1.30	7.01***
17:00–17:59	9.41	1.32	7.13***
18:00–18:59	7.41	1.35	5.46***
19:00–19:59	5.90	1.37	4.29***
20:00–20:59	5.62	1.39	4.04***
21:00–21:59	4.12	1.40	2.93**
22:00–22:59	1.59	1.45	1.10
23:00–23:59	2.54	1.49	1.70

Next, we applied a Linear Discriminant Analysis (LDA) to assess whether we could predict the diagnosis of patients based on gender age, and time of admission. For this analysis, we split the data in a train and test datasets. We trained the LDA model with 85% of our dataset and tested it on the remaining 15%. The results are presented in Table 2. Overall, the model showed modest results, with an accuracy of 44% (CI: 43, 45%) and Kappa of 17%. The model performed best classifying patients with the F1 diagnose, with a sensitivity of 84% and specificity of 41%. We compared the accuracy of the model with a second one which only included gender and age as predictors (supplementary table 2). We observed in the second model an accuracy of 43% (CI: 42, 45%) and Kappa of 16% (with a sensitivity of 84% and a specificity of 39% for the F1 diagnosis).

**Table 2:** Accuracy metrics of LDA predicting the diagnosis of patients based on age, gender, and time of admission at the hospital

	Diagnostic Group								
	F1	F2	F3	F4	F5	F6	F7	F8	F9
Sensitivity	0.84	0.05	0.48	0.0	0.0	0.11	0.0	0.0	0.0
Specificity	0.41	1.0	0.79	0.99	1.0	0.97	1.0	0.99	1.0
Pos. Pred. Value	0.46	0.0	0.41	0.14	0.0	0.34	0.0	0.0	0.0
Neg. Pred. Value	0.81	0.84	0.83	0.88	0.98	0.94	0.98	0.99	0.99
Prevalence	0.37	0.16	0.23	0.12	0.02	0.07	0.01	0.01	0.0
Detection Rate	0.31	0.01	0.13	0.0	0.0	0.01	0.0	0.0	0.0
Detection Prevalence	0.69	0.02	0.27	0.0	0.0	0.04	0.0	0.0	0.0
Balanced Accuracy	0.62	0.52	0.63	0.5	0.5	0.58	0.5	0.5	0.5

## Discussion

Regarding our first research objective, the conducted analyses showed that a large amount of psychiatric admissions occurs out of core time: on working days 24.9% of admissions take place out of core working time, on weekends/public holidays this percentage even rises up to 46.2%. These differences might (at least in part) be explained by the fact that admissions on weekends/public holidays per se are to be regarded as unscheduled and emergency-related. From an overall point of view, it can be stated that on workdays most patients seek psychiatric admission in the late hours of the forenoon with a peak at 10.01–11.00 a.m. However, on Saturdays/Sundays/public holidays the staff on duty has to face a consistent admission activity during the whole afternoon without clear peaks but stretching into the late evening hours without a steep decline (see Fig. 1). These findings are well in line with observations derived from a large study investigating psychiatric admissions on weekends vs. weekdays in Taiwan [9]. In this study, 661′709 acute psychiatric admissions between 1996 and 2012 were included based on the National Health Insurance Research Database, a proportion of 82′450 were weekend admissions. The patients with weekend admissions tended to be younger and the proportion of patients with schizophrenia, bipolar affective disorder, substance use disorder, and compulsory hospitalization were higher. Weekend admissions were associated with a shorter length of stay (30.3 days vs. 33.3 days, *p* < 0.001), lower inpatient morality rate (0.07% vs. 0.11%, *p* = 0.007), but higher readmission rate (26.8% vs. 25.3%, p < 0.001).

Several reasons may account for the difference in the distribution of admission times between weekdays and weekends in our own study. Beside the fact, that there are no elective admissions on weekends, one may speculate, whether the delayed distribution of admissions on weekends reflects the more “natural” course of the need of in-patient hospital care, whereas the distribution on weekdays is confounded by social and institutional factors. Independently from the reasons for the delayed distribution of admissions on weekends, our finding clearly indicates that the treatment of psychiatric conditions on a 24–7 level requires a consistent provision of adequate spatial and personnel resources or – in case of several neighboring small treatment units – a clever distribution of treatment responsibilities, provision of sufficient local inpatient capacities and advanced communicational strategies with allocating emergency service providers.

Regarding our second research objective we did not observe that patients that are admitted as emergency out of core time have a longer length of stay in the hospital than those, who come during the core hours. In contrary, patients arriving out of core time have shorter stays in the hospital. This unexpected result is probably mainly due to the use of “length of stay” as surrogate parameter for “condition severity”. Emergency admissions during the night occur mostly in acute patients, who are at the moment of admission severely ill, e.g. intoxicated, in a delirant state, suicidal or in an acute crisis. However, these acute pathologies improve typically much faster than the symptoms of chronically ill patients, who are typically admitted regularly during the day. Thus, for the comparison of disease severity between acute emergency admissions out of core time and planned admissions during the day, length of stay is probably not a very useful parameter. Moreover, there is a very high variability in the data. This effect may also be attributable to an (assumedly) lower quality of care during night hours due to the lack of time, qualification and competency for a thorough psychiatric evaluation. Based on the fact, that in most cases residents (still in professional training) are responsible for the provision of psychiatric care during night hours and seniors are mostly only available by telephone, a certain lack of competency to delay admission and/or provide ambulant care might also be contributing factors and serve as a possible explanation for the shorter duration of stay of these patients.

Therefore, even if there is a highly statistically significant relationship between time of admission and length of stay, our analyses also demonstrate that the information about time of admission is no relevant predictor for length of stay.

Similarly, “admission time” was no helpful predictor for the diagnostic classification of patients indicated by ICD-10 F-categories: the factors “age” and “gender” predicted 43% of the variability of the diagnoses, the addition of “admission time” only marginally improved our predictions to a total of 44%. Given the large cohort of more than 37′000 admission data sets the authors did not regard this gain of predictive value as clinically relevant in any way.

We are aware that the available dataset – albeit large, comprehensive and covering a time span of several years - is limited to one specific hospital serving as an exclusive provider of in-patient psychiatric care for a specific catchment area with 700′000 residents. Further studies will be needed to test, whether the data are representative, especially for more metropolitan areas or for psychiatric departments that are integrated in general hospitals. The authors are well aware, that the psychiatric care structure might be kind of a “special case” in Germany, where many large psychiatric hospitals are placed on the countryside with broad catchment areas instead of medium-sized psychiatric units in general hospitals such as in most other countries. Furthermore, it might also be mentioned as a limiting factor of our study, that due to long travel distances (attributed to the large catchment area of our hospital) planned admissions might have been delayed and therefore have arrived after core working hours distracting our analyses.

A further confounding factor is presumably the occupancy of the beds in the hospital. At times, when there are no free beds available, admissions might be further delayed as compared to days, when free beds are available.

A further important limitation of our study is the limited amount of available data per patient. For example, the hypothesis, that social circumstances such as homelessness etc. might have exerted an impact on the admission times, cannot be reliably ruled out based on our data as the cause of admission is an unavailable parameter in our dataset. Combining time of admission with more detailed information about the case (further details of admission, the patient’s state at admission or the leading symptoms at admission) might provide valuable insights that cannot be deducted from our data, as the respective information is not available in our dataset. This assumption is supported by previous investigations that found specific patterns for specific types of hospital admissions.

Eisenbach and colleagues [10] investigated patients after self-poisoning with suicidal intents during the years 2002 and 2004 in a cohort of 691 individuals and found a peak before midnight concerning the admissions. Variation with the day of the week was less clear in this investigation showing a peak incidence on Mondays without significant variations with monthly or annual cycles. The frequency of parasuicides was associated with “bad weather”. No association of parasuicide incidences to the lunar cycle was observed [10]. In another investigation by Buckley and colleagues similar results were presented after analysis of 2215 patients after self-poisoning with a marked circadian variation with over 6% of all admissions occurring in each of the hours between 6 p.m. and 1 a.m. compared with less than 2% per hour between 5 a.m. and 9 a.m. [11]

The results of our study are in line with investigations derived from other medical fields (mostly intensive care units) such as a large study by Arulkumaran et al. prospectively investigating a study cohort of 195′428 unplanned admissions from 212 adult general critical care units in England, Wales and Northern Ireland during a 3 year’s period without detecting significant influences on patient mortality for unplanned admissions to adult general critical care units within the United Kingdom [12].

However, this effect seems to be very sensitive to selection biases: Mohammed et al. retrospectively investigated a cohort of all routinely collected acute hospital admissions in England, involving all patient discharges from all acute hospitals in England over a year (April 2008–March 2009) involving 1′535’267 elective admissions (91.7% admitted on the weekday) and 3′105’249 emergency admissions (76.3% admitted on the weekday). After case-mix adjustment, weekend admissions were associated with an increased risk of death, especially in the elective setting (elective Odds Ratio: 1.32, 95% Confidence Interval 1.23 to 1.41); vs emergency Odds Ratio: 1.09, 95% Confidence Interval 1.05 to 1.13) [13].

The strength of our analysis is the large and representative sample, as the analysis covers all admissions of a hospital over the course of several years that is the only provider of psychiatric in-patient treatment of a large catchment area. Thus, the descriptive analysis of admission times provides several important insights in the circadian distribution of psychiatric hospital admissions. As an example, patients suffering from an F2-diagnosis (psychotic diseases) tend to seek admission later on a circadian basis than patients suffering from affective disorders (F3-diagnosis). This finding is remarkable, as a cross-sectional epidemiological study did report similar sleep/wake schedules in patients with F2 and F3 diagnoses [14]. In multivariate models the authors showed that patients with a depressive disorder or a psychosis were more likely to be morning type, whereas patients with an anxiety disorder, addiction disorder or personality disorder were more likely to be evening type [14]. Another important observation is the higher number of F1-diagnosis-related admissions during night shift/Saturday/Sundays, which is very likely attributable to the higher percentage of acute intoxications in the evening hours and weekends. However, this might be a structural observation depending on the local health care infrastructure: in other cities (even in Germany) acutely intoxicated patients may be treated in somatic hospitals rather than psychiatric care units indicating the need for cross-sectional studies comparing data between different sites and highlighting the importance of referring persons and institutions as well as local infrastructural circumstances. These confounders are one of the reasons that prevent the authors from specifically interpreting the observed diagnosis-related fluctuations of admission times as shifts in circadian rhythms. However, the authors feel that the presented data might serve as a groundwork to generate hypotheses deserving further investigations in that direction.

### Outlook on future research directions

Taking together it can be stated that psychiatric patients seek admission to a high degree out of core time and it remains open for speculation if a higher degree of patients suffering from somatic diseases might communicate with the hospital before admission and present themselves in core working hours therefore. However, these comparative data are lacking in our analysis and remain a matter of debate in future investigations. Moreover, future investigations should not only base on administrative data but also take into account the perspectives of referring physicians and patients.

## Supplementary information

**Additional file 1 Table S1**. Linear regression with the predictors “age” and “gender” and with the dependent variable “length of stay”. Multiple R-squared: 0.01; F-statistic = 142.3 on 2 and 35,301 DF, *p* value < 0.001.

**Additional file 2 Table S2** Accuracy metrics of LDA predicting the diagnosis of patients based on age and gender.

## Data Availability

The authors confirm that all raw data and materials are available for readers upon request. Please contact Peter Kreuzer as corresponding author.

## Abbreviations

BD: Bipolar disorder
LDA: Linear Discriminant Analysis
