# Supplementary material for: Limited predictive value of admission time in clinical psychiatry
Source: BMC Health Serv Res. 2020 Nov 13;20:1041. doi: 10.1186/s12913-020-05806-1 (PMC7663873; doi:10.1186/s12913-020-05806-1)
Supplement: Supplementary file 1 — Additional file 1 Table S1. Linear regression with the predictors “age” and “gender” and with the dependent variable “length of stay”. Multiple R-squared: 0.01; F-statistic = 142.3 on 2 and 35,301 DF, p value < 0.001. [file 12913_2020_5806_MOESM1_ESM.docx]

**Supplementary Material**

**Table 1.** Linear regression with the predictors “age” and “gender” and with the dependent variable “length of stay”.

|  | Estimate | Std Error | T value |
| --- | --- | --- | --- |
| Intercept | 18.79 | 0.53 | 35.57*** |
| Gender | 4.77 | 0.36 | 13.411*** |
| Age | 0.09 | 0.01 | 8.63*** |
