# Supplementary material for: Limited predictive value of admission time in clinical psychiatry
Source: BMC Health Serv Res. 2020 Nov 13;20:1041. doi: 10.1186/s12913-020-05806-1 (PMC7663873; doi:10.1186/s12913-020-05806-1)
Supplement: Supplementary file 2 — Additional file 2 Table S2 Accuracy metrics of LDA predicting the diagnosis of patients based on age and gender. [file 12913_2020_5806_MOESM2_ESM.docx]

**Supplementary Material**

**Table 2:** Accuracy metrics of LDA predicting the diagnosis of patients based on age and gender.

|  | Diagnosis | | | | | | | | |
| --- | --- | --- | --- | --- | --- | --- | --- | --- | --- |
|  | F1 | F2 | F3 | F4 | F5 | F6 | F7 | F8 | F9 |
| Sensitivity | 0.84 | 0.0 | 0.5 | 0.0 | 0.0 | 0.16 | 0.0 | 0.0 | 0.0 |
| Specificity | 0.39 | 1.0 | 0.79 | 1.0 | 1.0 | 0.97 | 1.0 | 1.0 | 1.0 |
| Pos. Pred. Value | 0.45 | 0.0 | 0.4 | 0.0 | 0.0 | 0.34 | 0.0 | 0.0 | 0.0 |
| Neg. Pred. Value | 0.81 | 0.84 | 0.83 | 0.88 | 0.98 | 0.94 | 0.99 | 0.99 | 0.9 |
| Prevalence | 0.37 | 0.16 | 0.23 | 0.12 | 0.02 | 0.07 | 0.01 | 0.0 | 0.0 |
| Detection rate | 0.31 | 0.0 | 0.11 | 0.0 | 0.0 | 0.01 | 0.0 | 0.0 | 0.0 |
| Detection Prevalence | 0.69 | 0.0 | 0.27 | 0.0 | 0.0 | 0.03 | 0.0 | 0.0 | 0.0 |
| Balanced accuracy | 0.61 | 0.5 | 0.63 | 0.5 | 0.5 | 0.57 | 0.5 | 0.5 | 0.5 |
